# Women’s Knowledge of Local Plants and Their Gastronomic Heritage in Chitral, NW Pakistan

**DOI:** 10.3390/plants13192747

**Published:** 2024-09-30

**Authors:** Arfaa Sabbah, Arshad Mehmood Abbasi, Muhammad Abdul Aziz, Fahdah Falah Benhasher, Andrea Pieroni, Ali Abdullah Aldosari, Mansour K. Gatasheh, Muhammad Amin

**Affiliations:** 1 Department of Environmental Sciences, COMSATS University Islamabad, Abbottabad Campus, Abbottabad 22060, Pakistan; 2 Department of Geography and Environmental Sustainability, College of Humanities and Social Sciences, Princess Nourh bint Abdulrahman University, Riyadh 11671, Saudi Arabia; 3 Department of Environmental Sciences, Informatics and Statistics, Ca’ Foscari University of Venice, Via Torino 155, 30172 Venezia, Italy; 4 University of Gastronomic Sciences of Pollenzo, Piazza V. Emanuele II, 12042 Bra/Pollenzo, Italy; a.pieroni@unisg.it; 5 Department of Medical Analysis, Tishk International University, Erbil 4001, Iraq; 6 Geography Department, College of Humanities and Social Sciences, King Saud University, P.O. Box 2456, Riyadh 11451, Saudi Arabia; 7 Department of Biochemistry, College of Science, King Saud University, Riyadh 11451, Saudi Arabia

**Keywords:** women’s knowledge, ethnobotany, food diversity, traditional cuisines, food security

## Abstract

Women are the “guardians of the kitchen” and central to household food security, yet their role has never been studied across the Hindukush region or Pamiri Knot. This study explores the women’s knowledge (specifically from the Khowar (Kho) and Wakhi linguistic groups) of local food systems and determines their role in ensuring household food security and sustainability in the mountain regions of northern Pakistan. Based on in-depth semi-structured interviews with female informants in the Rech and Broghil valleys of upper Chitral, 91 different types of food products were recorded, including wild and cultivated species. *Eremurus stenophyllus* and *Allium barsczewskii* were the commonly utilized plant species, though distinct preferences between the Kho and Wakhi groups were noted. Prominent differences were perceived in using certain cultivated plants among the two ethnic groups. For instance, Kho preferred plants like *Beta vulgaris*, *Zea mays*, and *Brassica napus* as indicated by the use reports, while Wakhi concentrated on *Thymus serpyllum*, *Zygophyllum obliquum* and *Papaver involucratum.* Both groups had shared dairy practices, but cottage cheese and curd were highly cited among Kho, while double-fermented curd and Qurut for Wakhi. The study recorded some new food uses for specific plants, such as *Atriplex hortensis*, *Carthamus tinctorius*, *Hylotelephium* spp., and *Saxifraga sibirica*. Cross-culture analyses revealed a mosaic pattern of homogenous and heterogenous trends based on reported food species of plants/animals and their use reports. Our findings emphasize the significant role of women in sustaining local food diversity, food sustainability, and the preservation and security of the local food systems, cultural legacy, and household food management. Therefore, inclusive research addressing their social, economic, and environmental issues must be conducted. Furthermore, policies must incorporate women’s traditional knowledge to build resilient food systems.

## 1. Introduction

Based on gender involvement, women tend to indulge more in practicing and preserving traditional food knowledge as they look for the most cost-efficient and eco-friendly alternatives for survival [1]. The traditional ecological knowledge (TEK) of women revolves mainly around survival [2] and supports the domestication and conservation of culturally significant plants and animal species specifically utilized in traditional food [3]. Indigenous women hold a decisive role in societal settings, and usually, they oversee the choice of foods in the household and promote traditional food products [4,5]. Women handle responsibilities mainly related to food production and consumption. Historically, they have been domesticating wild food species (plants and animals), choosing household food, preserving it, keeping food and seeds safe, and cultivating crops considering the best time and technique [2,6]. Moreover, women’s knowledge of wild edible plants, animals, and minor crops, as well as their cultivation, usage, preparation for food, and processing techniques, is predominantly passed down to future generations [7]. Therefore, their knowledge and skills grant them the most excellent position to preserve household food security and a sustainable food supply. Rural women often produce, manage, and market most food for their families and society. Hence, TEK about food processing, storing, and other crucial survival skills held by women plays a vital role in food preservation during seasonal food shortages. Historically, women can transform agricultural raw materials and animal wild food materials into nutritious and low-cost food products [8]. Hence, women are biodiversity keepers and critical contributors to household and regional food security and health care [6].

Women in mountainous regions are repositories of ecological and endangered plant and animal knowledge. Therefore, to achieve socioeconomic sustainability, the elimination of social and economic discrepancies and the promotion of women’s participation in decision-making is crucial [9]. Although women have information relevant to fostering food security, they are often underestimated regarding achieving and sustaining food security and traditional food for diets [10]. Unfortunately, women are disregarded as contributors in the TEK pool, mainly because their traditional knowledge is highly underrated in mainstream science. To create sustainable food systems and achieve food and nutrition security, it is essential to acknowledge the role of women’s Traditional Ecological Knowledge (wTEK) and their involvement in food supply chains. However, to some extent, their contribution has been considered significant in life sustenance and the management of biodiversity [4]. The inhabitants of Chitral profoundly depend on traditional means of subsistence, particularly women in this region who have historically served as the primary household food managers. However, their indigenous knowledge and contributions have been overlooked. Therefore, it is essential to explore the significance of traditional knowledge of Chitrali women in the context of food diversity, food culture, and traditional cuisines. Khowar and Wakhi are two major linguistic groups in the Broghil and Rech valleys, respectively. Therefore, the primary purpose of this study is to comprehensively document women’s Traditional Ecological Knowledge (wTEK), skills, and practices in traditional gastronomy within the Wakhi and Kho communities of upper Chitral. It also aims to evaluate the gastronomic legacies of the Wakhi and Kho people concerning food sustainability and explore strategies for its preservation and revitalization, considering contemporary food preferences and challenges.

## 2. Materials and Methods

### 2.1. Study Area

Chitral is the largest district of Khyber Pakhtunkhwa based on its geography and is covered with sky-touching mountains. It is located between 36.1113° N and 72.1416° E in the Hindu Kush Mountain range. The district is 14,850 square kilometers and is home to 447,362 people as per the 2017 census [11]. It has a northern and western border with Afghanistan, and the Wakhan Corridor separates it from Tajikistan’s Gorno-Badakhshan area. The present study conducted an extensive field survey to collect data from women of the Kho and Wakhi ethnolinguistic groups in the Broghil and Rech valleys of upper Chitral. The inhabitants of Chitral are known as “Kho” and are descendants of Aryans. Most have ancestral routes in Afghanistan, Tajikistan, China, and other Central Asian countries [12]. 

Broghil is the shortest route to Afghanistan, Central Asia, and China via Darwaza Pass and Broghil Pass [13]. This valley is between 36°45′ N and 73°30′ E at 3810 m from sea level. The total area of Broghil is 1348 km^2^, and it is home to ≈200 households [14]. Bounded by glaciers and mountains, the valley is ecologically significant because it is the gateway to the Indus Flyway for migratory birds [15]. The topography of Broghil is sloppy-uneven, including mountains, green plains, and valleys, and has around 3400 acres of peatland and lakes. The Wakhi and Sarikoli people in the Ismaili community live in Broghil. Wakhi cultural and family relationships across the Pakistan–Afghan border are common. The Wakhi are the major ethnolinguistic group in the Pamir region and have lived there for centuries (≈300–400 years), while the Sarikoli diaspora was originally from Taxkorgan county in Xinjiang province of China [16], and only one family is living in the last village of Broghil. During the field survey, we reached out to only one Sarikoli family living in the Lashkargaz village at Broghil. But it was discovered that their young generation no longer speaks the Sarikoli language. The last native speaker who could fluently spok Sarikoli passed away approximately 80 years ago, and there are currently no Sarikoli speakers remaining in Broghil although they declared themselves as Sarikoli. The Rech valley is 2237 m from sea level in Tehsil Torkhow of upper Chitral. The area is inhabited by Kho people, one of the dominant linguistic groups who speak Khowar. The entire population was around 4634 according to the 2003 census; however, according to a report, the current population is nearly 9000 [17]. Rech holds immense importance due to its strategic location; it shares borders with Yarkhoon and Ujnu from the East and South. Moreover, the valley is bordered by the Wakhan strip of Afghanistan. Rech has been under the spotlight due to the probable gas pipeline route between Tajikistan and Pakistan through the Anoshah pass across the Wakhan strip [18]. The Area falls under the sub-alpine category with mixed vegetation containing trees, shrubs and herbs. Rech valley has a mixed ethnicity regarding religious practices; the population belongs to the Sunni and Ismaili sects of Islam.

### 2.2. Field Survey and Data Collection 

**Figure 1:**
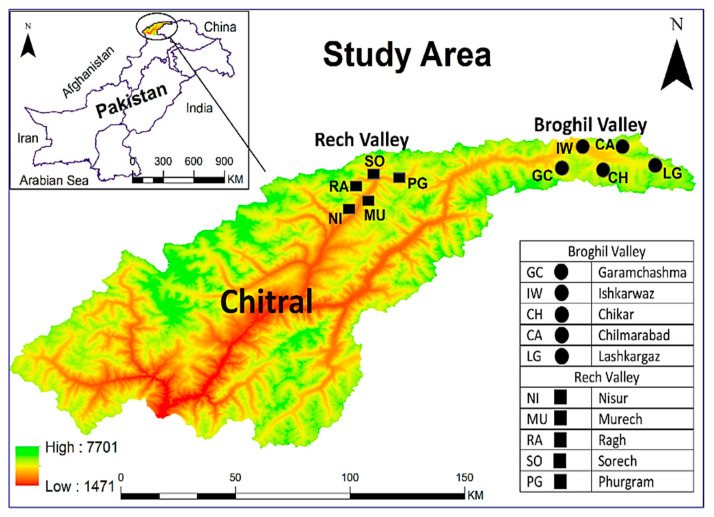
Map showing study sites in the Broghil and Rech valleys.

A comprehensive field survey was conducted from November 2023 to June 2024 in different areas of Broghil and Rech valleys, as illustrated in Figure 1. Rech is more accessible compared to Broghil valley, which made an additional visit possible. The first field survey in Rech was conducted during November and December 2023, with a follow-up visit in May and July 2024. In contrast, Broghil, being a remote valley, is only easily accessible between May and August. The challenging road conditions, heavy snow cover, and glaciers particularly during winter, limit access to Broghil. Therefore, brief field visits in different villages of Broghil were made in May and June 2024. During the field survey, five localities, namely, Lashkargaz, Chilmarabad, Chikar, Ishkarwaz, and Garam Chashma, were targeted in the Broghill valley, and five locations, viz. Nisur, Murech, Ragh, Sorech, and Phurgram, were the significant settlements that were visited in the Rech valley. Wakhi and Khowar are the two main linguistic groups in upper Chitral, which were the targets during the field survey. 

Before the field survey and interviews, the participants verbally agreed to interviews and photos, following the field ethical principles advised by the International Society of Ethnobiology [19]. The study employed semi-structured interviews, focus group discussions, and transect walks for seamless data collection following the previously described methodology by Aziz [20]. Verbal consent was obtained from all respondents to share data and photographs. 

The first author (Arfaa) is a female belonging to upper Chitral and familiar with the cultural setting of the area. She is a Khowar speaker and can speak/understand the Wakhi language and has direct assess to female respondents of the study area. The primary goal of the data collection was to compile details regarding plant- and animal-based traditional cuisines and recipes of wild, farmed, and domestic food sources in the study area. As shown in Table 1, 75 interviews were conducted through random informant selection in five locations, each in Rech and Broghil valleys. 

**Figure 2:**
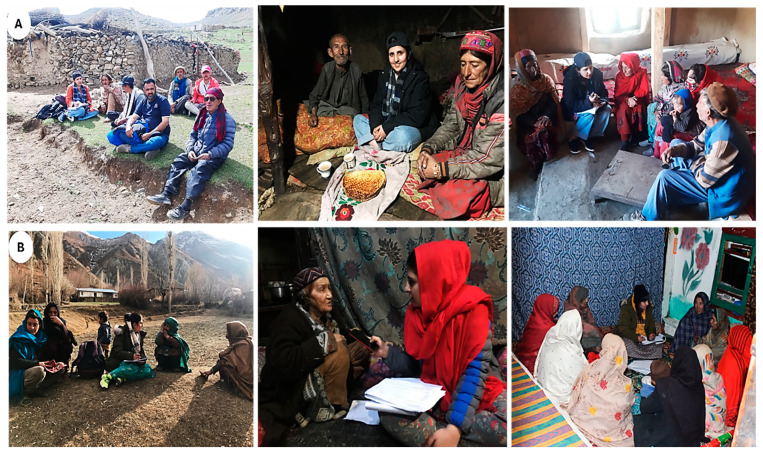
Field surveys and data gathering from key and general informants. (**A**) indicates Wakhi informants of Broghil valley, and (**B**) indicates Kho informants of Rech valley.

As illustrated in Figure 2, the interviews were divided into two categories based on age, experience, and expertise: general and key informants. Our study was underpinned by three robust data collection approaches, each serving a unique purpose and contributing to the richness of our findings.

- *Key Informant Interviews (KIIs):* Women 50 years and older with a solid understanding of traditional knowledge of the local food system participated as key informants (KIs). The snowball sampling technique was used to choose these KIs, and interviews were conducted utilizing well-versed semi-structured questions [20]. Information regarding crucial animal-based food supplies and wild and cultivated food plant species was the focus of the questions. The interviews highlighted culinary uses of plants such as vegetables, snacks, teas, seasonings, and traditional food preparation methods and shared information on dairy products.
- *General Informant Interviews (GIIs):* The general informant interviews were conducted with female community members of different age groups (15 to 40 years) to collect data on their food knowledge in general and traditional food knowledge in particular. The data gathered through general interviews evaluated young women’s understanding of food preferences and social eating trends.
- *Focused Group Discussions (FGDs):* The FGDs (*n* = 5) were held in each village as a supportive/additional activity to ensure the validity and authenticity of information provided by key and general informant not to be counted. Some glimpses of interviews from general informants and group discussions with Wakhi and Kho are given in Figure 2A and B, respectively. The FGDs served multiple vital roles in the research process as they validated and cross-verified information obtained from key informants, ensuring data accuracy and reliability. Moreover, FGDs facilitated in-depth exploration of research topics. Thus, by bringing together participants with varied insights and experiences, FGDs uncovered different facets of the subject, revealing nuances often missed in individual interviews. Additionally, FGDs provide context to the information collected, helping researchers understand how individual perspectives relate to group dynamics [21].

Since the Indigenous languages of Khowar and Wakhi were used for the data collection, the local names of every plant species were recorded and confirmed for accuracy several times. Notepads, voice recorders, and cameras captured data and field pictures of food plant species. All data were gathered using local linguistics and translated into English.

Edible plant species collected during the field survey were identified by expert taxonomists and cross-referenced with the Flora of Pakistan [22]. The nomenclature and botanical families of the identified plant specimens were verified using the international plant species database, The World Flora Online (https://www.worldfloraonline.org/). Mostly the voucher specimens were collected in flowering stage along with field photographs, and after processing (drying and mounting) were deposited in the herbarium at COMSATS University Islamabad, Abbottabad Campus, for future reference.

### 2.3. Data Analysis 

Qualitative and quantitative data were analyzed to comprehend the importance of women’s traditional ecological knowledge, specifically on upper Chitral food diversity, culture, and systems. The relative frequency of citation (RFC) was calculated to determine the relative cultural value of the reported food resources from the study area, as reported earlier by Aziz et al. [14], using the formula given below.

$$RFC=\frac{FC}{n}\times 100$$

where *FC* is the number of informants stated for food use of a particular plant, and “*n*” is the overall number of informants. 

The use reports (URs) for each category, viz. vegetables, flavoring, spices, snacks, tea, etc., were calculated using the formula as reported by Tardio and Pardo-de-Santayana [23].

$$\sum_{u=ui}^{uNC}\sum_{i=i1}^{iN}URui$$

Firstly, the URs of all the informants (from *i*1 to *iN*) within each use category were added, and then all the URs of each use category (from *ui* to *uNC*) were summed up. 

Moreover, we also applied Kruskal–Wallis test to compare the reported uses of food plants between the two groups. The Kruskal–Wallis test is a non-parametric method used when the assumptions of normality are violated, particularly for comparing more than two independent groups. This test evaluates whether the medians of the groups are significantly different or not. The Kruskal–Wallis test statistic (*H*) was calculated with the help of online software (https://www.socscistatistics.com/tests/kruskal/default.aspx) accessed on 20 August 2024, using the formula given below:

H = (12/(N(N + 1)) × (∑*T*^2^/*n*) − 3(*N* + 1)

where *N* represents the total number of observations across all groups and *n* represents the number of observations in each individual group. *T*^2^ refers to the sum of ranks for the group, which are squared before being divided by the number of observations in that group.

The study employed proportional Venn diagrams to visually represent the similarities and differences between the Kho and Wakhi groups. Venn diagrams were drawn using freely available software at http://bioinformatics.psb.ugent.be/webtools/Venn/ (accessed on 5 July 2024). Microsoft Excel was used to organize and display the results, utilizing the numerical data and our interpretive conclusions.

## 3. Results and Discussion

### 3.1. Diversity of Food Plants and Their Uses

The traditional food systems of the researched communities consist of diverse wild and cultivated food plants and wild and domesticated animals, along with their derived food products. The two valleys are an important ecological niche and place for growing essential food plants. Among the two groups, 65 plant species belonging to 24 families were used as food (Table 2). Legumes were majorly recognized; therefore, Fabaceae was the leading botanical family, with seven food plant taxa, followed by Lamiacea and Brassicacea, each with six species mentions. Since legumes are considered an essential crop in this mountain region, they are stored and important in food insecurities, especially in remote villages, where the connecting routes are broken in wintertime due to heavy precipitation (snowfall and rainfall), flash flooding, and landslides. As a leafy vegetable, the botanical taxa of the Amaranthaceae family are viable crops for the Kho and Wakhi communities, and similar food ingredients were also reported in other local communities in Chitral and Gilgit-Baltistan. In Rech valley, people used to consume fresh or dried fruits for winter, a part of the traditional system in wintertime when food is scarce. The concept of taste and flavor is highly famous among the Kho and Wakhi communities, and people quoted the Amaranthus as one of the famous vegetables that grow among the cultivated crops as a weed and are locally consumed as pot vegetables for their diverse taste and availability. The wide range of uses for the reported plants includes flavor and spices from the members of Apiaceae family, as well as edible species from the Asteraceae, Lamiaceae, and Brassicaceae families, which are well known for their aromatic properties across different regions, contributing to local food systems. These findings were consistent with earlier studies [14,20,24,25,26,27,28,29,30,31]. 

Kho and Wakhi women use mostly herbaceous plants (80%) collected in different seasons and habitats (Table 2). Flavors and textures play an important role in shaping the traditional food systems in these mountain territories. We found that the food plants were differently prepared using their various parts. Aerial parts (22 plant species) were frequently consumed, as shown in Table 2. Some plants were used in salads, while mostly the traditional food legacy consisted of cereal crops, mainly *Cenchrus americanus*, *Hordeum vulgare*, *Panicum miliaceum*, *Triticum aestivum* subsp. *spelta*, and *Zea mays*. However, both Wakhi and Kho use the aforementioned cereal crops in making different cuisines, but Kho cultivates these crops in Rech valley. But due to harsh weather in Broghil, Wkahi usually purchased cereals from local markets. 

Reported across the two valleys, both Kho and Wakhi communities reported that 37% of the plants were consumed as vegetables, followed by 10 (15%) consumed as snacks, 8 (12%) as spices in culinary art, 7 (11%) eaten raw as fresh salad, 6 (9%) used as flavoring agents, and 5 species (8%) as cereals (Table 2). Both communities reported that five plants collected in the wild were used in making recreational tea, and one plant (*Carthamus tinctorius*) was used for coloring food. Among the reported species, 32 species, including various spices, vegetables, and cereal crops, were cultivated, while 28 were collected from the natural environment (Table 2). Moreover, five vegetable species, viz. *Chenopodium album*, *Atriplex hortensis*, *Medicago sativa*, *Mentha suaveolens*, and *Zataria multiflora*, were reported as wild and cultivated. Some vegetables like *Allium barsczewskii*, *A. carolinianum*, *Chenopodium album*, *Buglossoides arvensis*, *Atriplex hortensis*, *Eremurus stenophyllus*, *Hylotelephium ewersii*, *Lepyrodiclis holosteoides*, *Malva neglecta*, *Medicago sativa*, *Rumex dentatus*, *Silene conoidea*, and *Zygophyllum obliquum* are harvested in their early stages and eaten as cooked vegetables. Female informants of Wakhi in Broghil valley stated that they consume the young leaves of *Taraxacum campylodes* as a vegetable and avoid eating their mature leaves because of their unpleasant taste. *Brassica rapa*, locally called “Chirogh”, does not mature due to the harsh climate conditions; thus, the young leaves are harvested and consumed as vegetables, whereas in warmer regions, the same species is utilized for oil production.

In Rech, the local food system is more diverse; in fact, there are influences of external food ingredients compared to Wakhi, as we have observed that Kho used more spicy food than Wakhi. Living in high mountain pastures, the Wakhi group, on the other hand, has a more spartan culinary tradition due to limited resources. The Wakhi are more economically marginalized, living in the upper remote parts of the valley, where the winter season is very harsh. They usually prefer food that keeps them energetic; therefore, the “hot/warm food” concept could be considered for further research. Women in both communities reported that drying food ingredients is a reliable and widely used method for preserving plants, which are used as vegetables, spices, and teas for the off-season. Among the Kho community, *Anthemis nabataea*, *Artemisia absinthium*, *Cuminum cyminum*, *Mentha longifolia*, *M. suaveolens*, *Saxifraga sibirica*, *Trigonella gladiata*, *Zataria multiflora*, and *Ziziphora clinopodioides* are often dried and used as spices during winter to enhance the taste. Wakhi reported the sun-drying of *Papaver involucratum* and *Thymus serpyllum* for tea, specifically in winter. Participants reported that fresh fruits, tubers, and vegetables are buried underground at least 1 m and stored for a long time while covered in wheat or rice straw. This specific traditional method of storing and preserving food for a long time was named Sethyni and is famous among the Kho community of Rech valley.

Descriptive statistics have shown 1648 URs for the 65 recorded food plant taxa (Table 2). Vegetables were frequently quoted with the highest URs (596), accounting for 24 plants. Highly famous wild food plants used as vegetables were *Eremurus stenophyllus* and *Allium barsczewskii*, among both the communities having URs of 68 and 64, respectively. Likewise, 233 use reports (URs) of 15 plant species used as snacks were mentioned, with *Beta vulgaris* being the most reported, accounting for 35 URs. Additionally, 199 URs were recorded for 11 species used as flavoring agents, with *Allium cepa* leading at 34 URs. Additionally, 135 URs for fresh salad were recorded for 11 species, and 92 URs of tea for nine species were listed, whereas the rest of the food categories had fewer than 50 URs of reported plant species.

**Figure 3:**
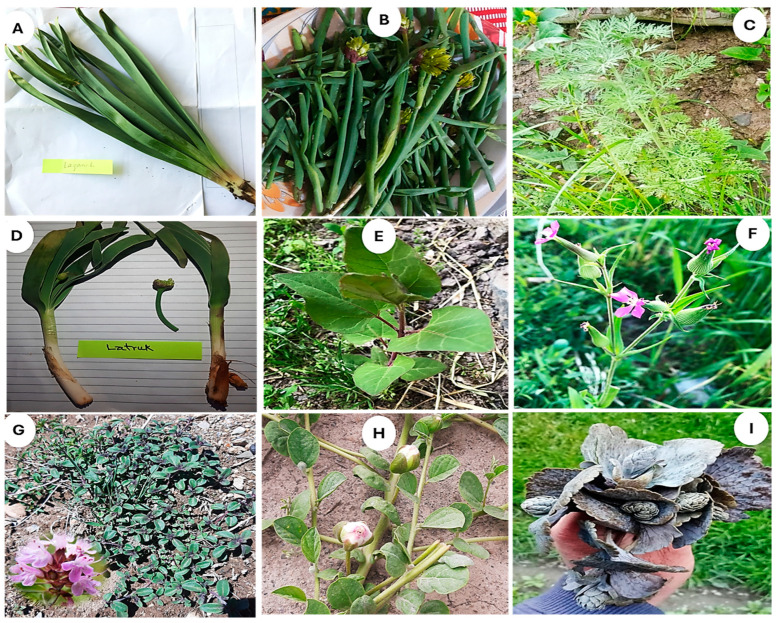
Most of the cited and unique food plants in the upper Chitral region are based on women’s traditional knowledge. (**A**) *Eremurus stenophyllus*, (**B**) *Allium barsczewskii*, (**C**) *Artimesia absinthium*, (**D**) *Allium carolinianum*, (**E**) *Atriplex hortensis*, (**F**) *Silene conoidea*, (**G**) *Thymus serpyllum*, (**H**) *Capparis spinosa* (**I**) *Hylotelephium* spp.

The RFC of the reported food plant species (Table 2) reflects their importance and prominence in the local food system. Some of the most cited and unique food plant species of upper Chitral reported by Kho and Wakhi women are shown in Figure 3. Among the common plant species, *Eremurus stenophyllus*, with an RFC of 0.880, was at the top, followed by *Allium barsczewskii* with 0.853 RFC (Figure 3A,B). Likewise, *Artemisia absinthium*, *Allium carolinianum*, *Lepyrodiclis holosteoides*, and *Atriplex hortensis* had more than 70% citations. Moreover, *Silene conoidea*, *Thymus serpyllum*, and *Capparis spinosa* exhibited more than 50% RFC. The Kho women reported *Beta vulgaris*, *Zea mays*, *Brassica napus*, and *Artemisia absinthium* as the topmost cited plant species with the highest RFC (0.87, 0.85, 0.82, and 0.77, respectively). Likewise, *Thymus serpyllum*, *Zygophyllum obliquum*, and *Papaver involucratum*, with RFC 0.60, 0.40, and 0.30, respectively, are quoted as the most cited food plants by the women of Wakhi in Broghil. Women have affirmed that they are mainly responsible for gathering, drying and preserving wild vegetables for winter or off-season. They gather these vegetables throughout the summer when they move to the high mountain pastures with their herds. 

### 3.2. Animal-Based Food Diversity in Upper Chitral

The current study reported ten animals, including livestock, wild birds, and wild animals, that contribute significantly to the food culture of Kho and Wakhi inhabitants (Table 3). This is the first-ever study emphasizing detailed documentation of animal-based food resources among the Kho and Wakhi communities of upper Chitral. The animals belong to the Bovidae, Columbidae, Anitidae, and Phasianidae families. Among these, the Bovidae family was leading with five animal species, followed by three species in Columbidae, and the rest of the two families were represented by one animal species each.

**Figure 4:**
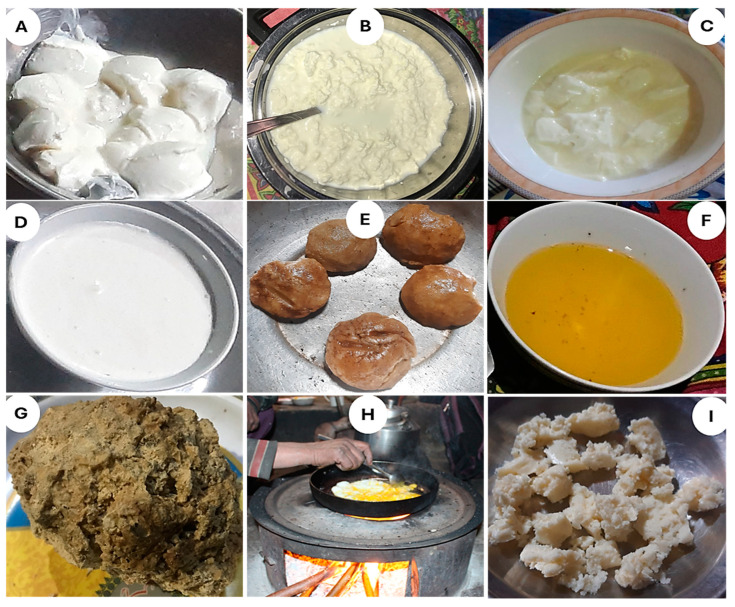
Most cited dairy products. (**A**) Macheer, (**B**) Phenak (**C**) Trin, (**D**) Mirk/Khomokh, (**E**) Qurut, (**F**) Zugh Rugon/Don, (**G**) Shut, (**H**) Ayukun, and (**I**) Maska/Khomrugon.

*Anas crecca*, *Bos taurus*, *Capra hircus*, *Capra sibirica*, *Ovis aries*, and *Streptopelia decaocto* were common animal species reported by the women informants of Khowar and Wakhi linguistic groups. *Bos grunniens* (yak) and *Columba leuconota* (snow pigeon) were exclusive to Broghil as they are most compatible with high alpine pastures and colder regions (as stated by a Wakhi informant). *Gallus domesticus* (hen) and *Columba livia* (rock pigeon) only exist in the Kho-populated villages (Rech valley). In Wakhi areas (Broghil), harsh weather and wild carnivores are significant reasons for the lack of domestication of hens and rock pigeon. A total of 16 different animal-based food products were reported by the women of Kho and Wakhi (Table 3). A significant chunk of the dairy products, including various types of milk and cheeses consumed in different ways. Among the typical products shared by both groups, Khombokh/Mrik Cheer/Xarj and Don/ Rugon (in Khowar/Wakhi) were the top cited products with RFC 0.74, 0.65 and 0.58, respectively (Figure 4). Phenak (cottage cheese) and Trin (curd) were unique dairy products for Kho, with a maximum RFC of 0.75 each. In contrast, Macheer (a double fermented curd) and Qurut (a hard cheese) were quoted frequently and obtained the highest RFCs of 0.80 and 0.65, respectively, reported by Wakhi women. The fat of yak, cow, goat, ibex, and sheep is used to make traditional cuisines.

### 3.3. Traditional Cuisines of Kho and Wakhi Communities

During the current study, we recorded 56 traditional food products among Kho and Wakhi linguistic groups in the study area (Table 4). These food products mainly consist of either plant- or animal-derived products that are readily available to the local communities in the area. The plant-based traditional cuisines make up around 46%. In comparison, animal-based food ingredients were only 9%, and this could be referred to as the difference in the available food ingredients in the Rech and Broghil valleys. A considerable portion (45%) of the traditional foods were derived from combining plants and animals. We have observed that the traditional diet of the researched communities mainly consists of dairy products like ghee, butter and fat, and the available fresh herbs. Both groups prefer major savory dishes with limited green flavoring and the addition of minimal spices. Most women respondents affirmed that people prefer salty and the least spicy foods with meat flavors. More than 70% of the respondents noted the health benefits of traditional food ingredients. Participants confirmed that in the Broghil valley, the conventional food ingredients are gradually changing as the invasion of food commodification is spreading in the area, devaluing the local food products and having negative health consequences. One of the participants among Wakhi added that commercially available food items may cause several diseases like gastric problems, kidney problems, and bone and joint disorders; therefore, traditional food ingredients are still a good choice for maintaining health. 

**Figure 5:**
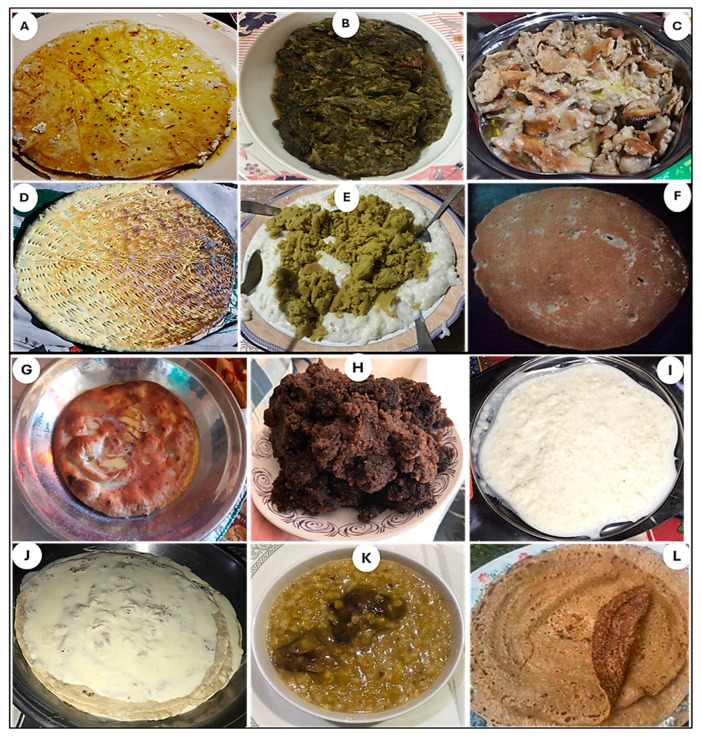
Most cited and unique traditional cuisines reported by Kho and Wakhi women. (**A**) Ghalmandi, (**B**) Shakh, (**C**) Qurutaab, (**D**) Thonikcha, (**E**) Sanabachi/Bat, (**F**) Khesta shapik/Khesta khech, (**G**) Shindet, (**H**) Shoshp, (**I**) Cheer grinj/Sheer bronj, (**J**) Cheera Shapik/Xharjkhech, (**K**) Laxek, and (**L**) Rishok.

As illustrated in Figure 5A,B, *Ghalmandi* and *Shakh* are the signature cuisines of Kho speakers in Rech, with the highest RFC (≥80%). The *Ghalmandi* is rich in nutrients and is considered the epitome of taste across Kho culture. It is prepared using traditionally toasted tortillas locally called “Phulka” and cottage cheese. Cottage cheese is added to finely chopped coriander leaves and salt, and a paste is later prepared and sandwiched between tortillas. The dish is topped with sizzling butter and served. The *Shakh* is prepared using green or dried vegetables. Vegetables are boiled and then chopped and fried in butter/desi ghee, along with onions and tomatoes, and other seasonings and spices are also added to enhance taste. The fried paste is added to some water and is boiled. After a good boil, a flour paste locally called “Khanjur” is added, and the dish is simmered until it is done. Another substantial Kho dish cited as “*Laxek*” with RFC 0.62 is prepared from soaked wheat grains, which are later coarsely ground. The ground wheat grains are added to the meat stew and boiled until done. 

In contrast to Kho, Wakhi inhabiting Broghil has the rare cultivation of cereal crops, vegetables, and fruits. Hence, their traditional cuisines are majorly shaped by dairy products. The Wakhi cuisines are simple but rich in high-energy ingredients such as fats and proteins to match the challenging life-sustaining activities in the pastures of Broghil. As shown in Figure 5C, *Qurutaab* is the most highly cited (0.74 RFC) cuisine of Wakhi. *Qurutaab* is made from *Qurut* (a hard local cheese illustrated in Figure 4E), soaked in water and later a toasted bread (*Dildongi*), and desi ghee are added and served. Likewise, *Thonikcha*, *Dildongi*, *Brat/Qomuchdoni*, and *Ptoq* (all having RFC ≥ 0.6) are different types of bread made of wheat consumed by the Wakhi women. Hence, wheat as a noteable ingredient plays a significant role in making local breads and traditional recipes in both valleys. However, in Broghil, due to limited and inadequate production, Wakhi purchase wheat from local markets of upper and lower Chitral. 

*Thonikcha* (Figure 5D) is made with freshly kneaded wheat dough, desi ghee, and milk. The flatbread (*Thonikcha*) is toasted in a traditional mud oven called a “*Thanoor”*. Qurut is directly splashed in water or milk to enhance the taste while roasting *Thonikcha* in *Thanoor*. The *Dildongi* and *Ptoq* are made from fermented dough kneaded with locally cultured yeast. Flatbread is rolled out, put into the mud oven, and roasted slowly. *Dildongi* is of regular size, while Ptoqs are small and usually confined to preparation during a wedding. *Shindet*, made of Dildongi, ghee, and milk, is a unique traditional Wakhi cuisine. The freshly baked/toasted *Dildongi* is placed into a serving pot with milk and seasoned with sizzling hot desi ghee. In addition, *Shindit*, some unique 

Traditional cuisines reflect the way of life and environment that the people of an area share. Kho and Wakhi have 16 similar traditional cuisines (Table 3), signifying a common culinary heritage and shared culture. Among these, “*Sanabachi*” in Khowar or “*Bat*” in Wakhi was the most cited, with an RFC of 0.92 (Figure 5E). The *Sanabachi* or *Bat* is made of desi ghee and wheat flour, both served alone and incorporated with other cuisines. The wheat flour is fried in butter or ghee until it attains the required consistency and light brown color. The Kho adds *Carthamus tinctorius* as a coloring agent, while the Wakhi does not use any coloring agent. *Khesta Shapik* (Khowar) or *Khesta Khech* (Wakhi) is also one of the most cited everyday dishes of both communities (≥70% of respondents). It is a type of fermented bread prepared from a dough batter with a particular consistency, fermented using local yeast cultures.

### 3.4. Cross-Cultural Comparison of the Local Food Heritage

**Figure 6:**
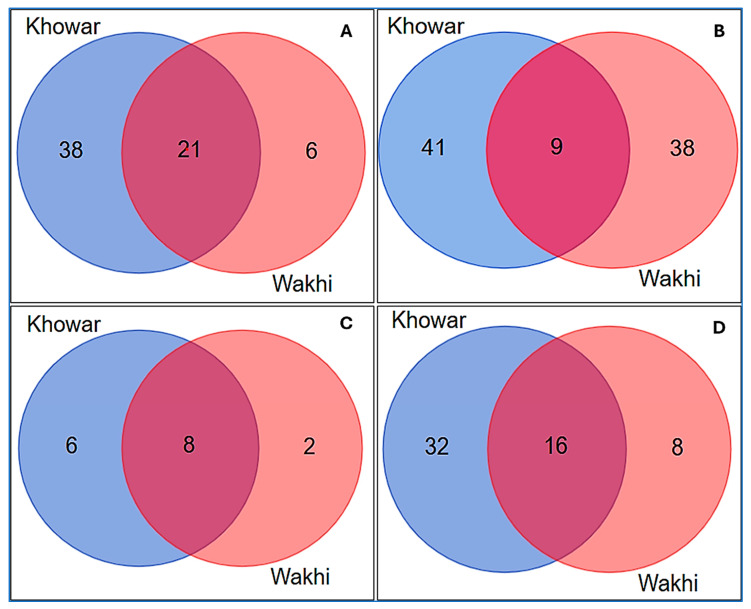
Venn diagrams illustrate cross-cultural analysis of (**A**) food plant diversity, (**B**) use reports, (**C**) animal-based products, and (**D**) traditional cuisines cited by Kho and Wakhi women in upper Chitral.

A cross-cultural comparison, as illustrated by Venn diagrams (Figure 6), was made based on food plants reported by Kho and Wakhi women in the Rech and Broghil valleys of upper Chitral. Both communities have different lifestyles and ecotones, so they depict relatively homogeneous trends in resemblances. As shown in Figure 6A, 21 species that account for 32% of the reported food plants in upper Chitral were commonly used by Kho and Wakhi. The Kho women reported 38 food plants, attributing 58% of the listed species in Table 2, owing to the plant diversity in the area. However, due to minimal vegetation in the valley, the Wakhi community statistics show only six unique food plant species that account for only 9% of the reported food plant taxa. 

Lifestyle and culinary traditions also influence the number of citations as the flavoring and spices are more common among the Khowar-speaking group, while Wakhi prefers simple and savory cuisines. The food plants mentioned exclusively by the Kho women were *Artemisia absinthium*, *Benincasa fistulosa*, *Beta vulgaris*, *Brassica napus*, *B. oleracea*, *Buglossoides arvensis*, *Capparis spinosa*, *Carthamus tinctorius*, *Cenchrus americanus*, *Atriplex hortensis*, *Cucurbita maxima*, *Ferula narthex*, *Hylotelephium* spp., *Juglans regia*, *Lathyrus oleraceus*, *Lens culinaris*, *Lepidium ruderal*, *Malus domestica*, *Medicago sativa*, *Mentha longifolia*, *M. suaveolens*, *Nasturtium officinale*, *Panicum miliaceum*, *Prunus amygdalus*, *Prunus armeniaca*, *Prunus avium*, *Pyrus bourgaeana*, *Salvia rhytidea*, *Saxifraga sibirica*, *Silene conoidea*, *Solanum lycopersicum*, *Solanum melongena*, *Trigonella gladiata*, *Vicia faba*, *Vigna unguiculata*, *Zataria multiflora*, *Zea mays*, and *Ziziphora clinopodioides.*

Whereas the Wakhi women mentioned *Brassica rapa, Chenopodium foliosum, Papaver involucratum, Taraxacum campylodes, Thymus serpyllum*, and *Zygophyllum obliquum* were unique to them. Nevertheless, *Allium barsczewskii, A. carolinianum, A. cepa, Anthemis nabataea, Chenopodium album, Berberis vulgaris, Coriandrum sativum, Cuminum cyminum, Daucus carota, Eremurus stenophyllus, Hippophae rhamnoides, Hordeum vulgare, Lactuca sativa, Lepyrodiclis holosteoides, Malva neglecta, Pisum spp., Raphanus raphanistrum, Rheum ribes, Rumex dentatus, Solanum tuberosum*, and *Triticum aestivum* were reported by both the targeted groups.

The food culture and customs shape the uses of plant species in multiple ways, so the study data reflect multiple uses for each plant cited by the informants. The Venn diagram (Figure 6B) indicates heterogeneity in use reports of the quoted food plant species. A total of 41 (46%) URs were unique to the Kho, and 38 (43%) were exclusive to the Wakhi, while 9 (10%) were shared between both groups. The Kruskal–Wallis test statistic (H) was found to be 0.69, with a corresponding *p*-value of 0.41. These results indicate that there is no statistically significant difference in the number frequency of citation for the respective use reports of the quoted food plants among the two groups (*p* > 0.05). The results (*p* = 0.41) suggest that both groups tend to retain a similar pattern of frequency for the reported food plants. This lack of significant difference may reflect shared ethnobotanical knowledge, similar ecological conditions, or both. Therefore, while the groups may have distinct cultural identities, their reliance on and selection of food plants does not appear to differ statistically.

Relatively, Kho women reported more diverse uses because of the diversity of food plant species, including herbs, shrubs and trees in Rech valley. However, Wakhi inhabits high-elevation pastures, has limited vegetation, and depends on animal-based food. A comparative assessment of animal-based products among the Kho and Wakhi linguistic groups is demonstrated in Figure 6C. Out of sixteen different types of products, eight products (50%) were common in both linguistic groups. In contrast, six products (37%) were exclusively reported by Kho women and only two (12.5%) that are Qurut and Macheer, were reported by the Wakhi group. More animal products are available in the Kho community than in the Wakhi community. Nonetheless, a notable overlap indicates everyday activities and mutual interactions between the two linguistic groups as both are exogamous and have inter-marriages. The similarity also reflects adaptations to similar climatic conditions; thus, certain animal products are more readily available or particularly suited to the lifestyle and consuming patterns. As demonstrated in Figure 6D, the Kho community in Rech had more diverse traditional cuisines (TCs) than Wakhi living in Broghil pastures. Both linguistic groups commonly reported 16 TCs that account for 28.5% of the total, while 32 (57%) were specific to the Kho and 8 (14%) to the Wakhi. This variation is mainly due to the availability of various cultivated cereal crops, i.e., rice, barley, millet, wheat, etc., along with different lentils, potatoes, fruits, and various cultivated and wild vegetables in Rech valley inhibiting by the Kho.

### 3.5. Novelty in Food Diversity of Upper Chitral 

The research biasedness to women’s participation in ethnobiological studies is high, particularly in religious and culturally rigid communities, as the social setting is a mirror image of a typical patriarchal society. Therefore, current knowledge of traditional food resources, mainly possessed by women in mountain regions, is more challenging to explore. As suggested by Aziz et al. [28], a deeper insight into the women’s traditional ecological knowledge (wTEK) on food resources in mountain regions of Pakistan is imperative. In the previous studies focused on food and medicinal plants in Chitral and its surrounding areas, gathered data were mainly from male informants, with an insignificant proportion of women. However, the current study has thoroughly examined and documented wTEK regarding the diversity of food and food culture, specifically in the high mountain areas of Chitral. Specifically, the present study is a groundbreaking investigation emphasizing the wTEK possessed by Kho and Wakhi women in terms of local food diversity and the food system. 

Plenty of research from this region has been reported focusing on edible and medicinal plants. However, to our knowledge, the diversity of animal-based food and traditional cuisines has yet to be reported from this region. Table 2 mentions 58 plant species (89%) that have been reported as food in Chitral’s ethnobotanical literature and surrounding areas [14,26,27,28,33,34,35,37,39,43,44]. However, four food plant species, including *Atriplex hortensis*, *Carthamus tinctorius*, *Hylotelephium* spp., and *Saxifraga sibirica*, constituting 6% of the recorded taxa, were reported for the first time as food plants from this region. 

*Hylotelephium* (Figure 3I) is a rare species that is used as a fresh vegetable by the Kho community of Rech valley due to its unique taste and health benefits. This plant has been reported as a perennial herb from temperate regions of Shangla and Jammu and Kashmir regions, Pakistan [45,46]. However, to our knowledge, *Hylotelephium* had never been reported as a food plant before from Pakistan, but in India, Devi et al. [47] reported *Hylotelephium ewersii* as a vegetable. However, medicinal uses of *Anthemis nabataea*, *Chenopodium foliosum*, *Saxifraga sibirica*, and *Trigonella gladiata* have already been reported by various workers from this region and surrounding area [26,33,34,35,37,39,43,44], but their use as food has never been reported before. 

**Figure 7:**
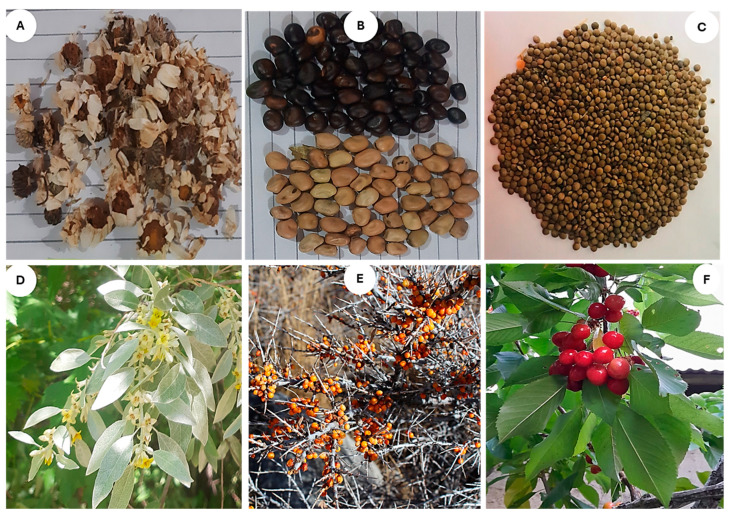
Unique and economically important plant species reported by Kho and Wakhi women. (**A**) *Anthemis nabataea*, (**B**) *Vicia faba*, (**C**) *Lens culinaris*, (**D**) *Elaeagnus angustifolia*, (**E**) *Hippophae rhamnoides*, and (**F**) *Prunus avium*.

The diversity in the plant part(s) used of some species creates a certain nuance in mountain gastronomy. For instance, lentil species are noteworthy in Kho food culture, particularly in Rech valley. *Pisum* spp., locally called “Kuchoon,” and *Lens culinaris*, called “Sirju” (Figure 7 B,C), have unique uses in Kho culture, reflecting their distinctive culinary significance and exceptional traditional knowledge and practice. 

The flour of these lentils is used to prepare a traditional Kho dish called Leganu. Similarly, *Pisum* spp. has the unique feature of producing two-color (black and white) seeds in a single pod. The seeds’ flour of *Pisum* is used in preparing traditional Kho bread, locally called Kuchunai.

Food plants, particularly wild edible plants, have been incorporated into traditional health systems for their health and therapeutic properties. People in remote areas with limited access to basic health facilities exploit natural healing and curative methods, creating a history of using food as medicine. Similarly, in the study, significant herbs were reported to be used frequently due to their health benefits. Most informants mentioned *Allium barsczewskii* as one of the most used herbs due to its effectiveness against joint and muscle pain. In addition, some of the informants also noted that it helps to maintain normal blood pressure. *Allium carolinianum* was reported to be effective for muscle pain, especially in the legs, as reported previously by Aziz et al. [31]. Still, the study accounts for its curative properties against hepatitis and high blood pressure. *Eremurus stenophyllus* was frequently cited as a wild vegetable and is highly energetic, as informed by the women informants, and thus used as a laxative. According to the informants, vegetables are the primary source of nutrition and are a vital part of mountain gastronomy due to their positive impacts and effectiveness against digestive problems.

*Anthemis nabataea* has many uses in traditional medicines in the study area, so it is an important part of curry and soup. It is highly aromatic and effective in treating fever and abdominal issues. It is also used as a major tea ingredient, which is taken orally to relieve gastric problems and is beneficial for jaundice.

*Saxifraga sibirica* locally known as Dromosoro has been reported as a general body tonic and is considered as a very important healing herb for backache, as reported by Haider and Qaiser [32]. The current study reports it as a flavoring agent in bread, which is used for its medicinal benefits among women to treat lower back pain and gynae issues. Similarly, *Artemisia absinthium* and *Cuminum cyminum* are also added to soup and other food curries due to their effectiveness against gastrointestinal problems and to lower blood pressure. *Ferula narthex* is used extensively as a snack to maintain blood pressure and improve digestion among Kho people of Rech during summers. According to the women respondents, milky latex from this species is extruded and dried, which is locally called Hing and is used for multi-purposes. For instance, it is taken orally to treat cardiovascular and gastrointestinal diseases, and to protect children from the evil eye. The latex is sold in markets and is normally available among elderly people. 

*Capparis spinosa* a vital wild shrub which has history of medicinal use in the mountain, is eventually merged into gastronomic practices of mountain communities. The Kho people have been using the flower buds of this species as key ingredient in the local soup Kaveerogh to treat typhoid and other inflammatory problems. Apart from the soup, it is a major flavoring agent for multiple local curries and dishes. The medicinal and therapeutic effectiveness against typhoid, diabetes, heartburn, and high blood pressure has already been discussed in the literature [33,43]. 

In the pastures of Broghil and Rech, usually cereal crops, especially wheat flour, are commonly used in making a variety of bread. With the heavy consumption of wheat, most of the people suffer from indigestion problems, mainly constipation. Therefore, they tend to use *Panicum miliaceum*, *Pisum sativum*, *Zea mays* and *Hordeum vulgare* flour instead of wheat flour for bread and tortillas.

In a nutshell, the traditional gastronomy has undergone evolution with the addition and deletion of certain natural ingredients based on their medicinal benefits. Our results indicate that in these remote valleys of the Chitral, women have been not only the guardians of the traditional kitchen but also have deep understanding of the local natural resources.

### 3.6. Socio-Economic Perspective of Local Food Resources in Upper Chitral

Some of the recorded plants have been reported to have high socio-economic values and are part of their subsistence economies and livelihoods across the two communities. *Elaeagnus angustifolia* and *Hippophae rhamnoides* (Figure 7E) are wild edible plants with high economic value and are sold in the local markets. For instance, the people of Kishmanja village of Broghil collect the fruits of *H. rhamnoides* and sell them at 500–700 rupees/kg. Similarly, local honeybee farms benefit from the *E. angustifolia* flowering season by producing honey of high worth (≈3500 rupees/kg) in the local and regional markets. Comparatively, Rech valley has a diverse range of fruit trees, making the food system stable as compared to Broghil. For instance, the multipurpose use of fruit trees like *Juglans regia*, *Prunus avium*, *P. armeniaca*, and a variety of apple trees are used as fuel, fodder, and timber by the inhabitants of Rech. 

**Figure 8:**
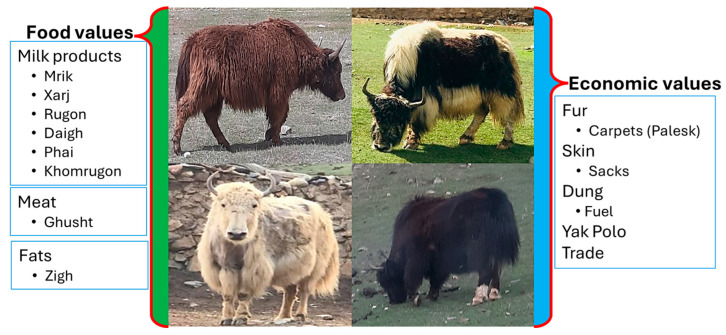
Food and socio-economic values of yak in upper Chitral.

Yak (*Bos grunniens*), the signature animal of high-altitude regions in Asia that can survive and tolerate harsh climatic conditions [48], is a fundamental source of food (meat, milk) to pastoralists (Wiener). Their importance to the Broghil communities is multifaceted in terms of culture, nutrition, and economy. Yaks have been vital in nomadic life due to their worthy contribution as food and a means of income generation. In Broghil, different breeds of yak, viz. black, brown, white, and mixed yak, are common and contribute significantly to socio-economic development and fulfilling the food requirements of the Wakhi community (Figure 8). 

The milk of Yak is used to make staple food in the Wakhi diet. Yak milk is processed to make butter, cheese, and yoghurt (Table 3), contributing to the community’s nutritional intake and local food culture. Another essential constituent is yak meat, which provides a consistent source of protein all year round, especially in the winter. Apart from meat and dairy products, the fur of yaks has been traditionally used in making local carpets (Palesk), and its skin is used in making sacks to store/preserve grains. Moreover, yaks are widely incorporated into local customs and ceremonies (yak polo), which gives them a significant cultural role. In addition, Yak dung is also used as a fuel source for cooking and heating to minimize reliance on outdoor fuel resources. According to [40], yak husbandry in Pakistan has been dramatically impacted by multiple factors, such as disease outbreaks, predation by wild animals, and insufficient veterinary treatments. Key informants in the Wakhi community have reported the same information regarding the decrease in yaks. Likewise, the decline in flora and pasture availability due to the changing climate in the northern rangelands of Pakistan is showing a rapid impact on yak herding [49]. One of the critical female respondents (75 years old) in Broghil highlighted a significant decrease in the number of yaks in the last two decades due to the excessive selling by Wakhi people in the local markets to fulfil their children’s market-based foods and educational requirements. The Wakhi people purchase flour, rice, and lentils twice a year from nearby marketplaces located at Upper Chitral, and for this purpose, they sell out their healthy yaks frequently. Therefore, the sustainable management and conservation of yak herding are essential to the resilience and survival of these high-altitude communities.

### 3.7. Kho and Wakhi Women’s Knowledge and Food Security 

Food and health are closely related, significantly impacting general well-being with diet variety and quality. One method by which women maintain family health is regulating food consumption among family members. This illustrates how women can produce spices and cook fancy meals for their families [50]. Since diet-related chronic diseases account for a significant portion of deaths worldwide, the topics of food security, food systems and food culture, and food and health are gaining more and more attention from ethnobiologists and ethno-pharmacologists. “Food medicine” refers to consuming various natural foods to achieve a therapeutic effect or prevent diseases [28]. 

Since Wakhi and Kho’s women have become accustomed to practicing traditional culinary methods, they have been significant in preserving and securing food. Their extensive understanding of local cuisines, cooking techniques, and preservation techniques ensures the year-round availability of healthful food. By preserving and transmitting traditional recipes, women’s traditional knowledge helps to provide a solid and diverse food culture in the study area despite climatic, economic, and environmental challenges. Women’s knowledge encompasses the selection of local cuisines and traditional food preservation techniques to ensure food availability and nutritious content throughout the year. Indigenous women’s proficiency in drying, fermenting, and performing other preservation techniques is fundamental for storing food in abundant seasons and guaranteeing a consistent supply in infertile ones. Some traditional preservation techniques were reported in this study, apart from just drying vegetables and fruits. For instance, Sethyni is a traditional way of preserving the Kho community’s tuber and root vegetables for winter. In this method, the ground is dug about 1–2 m, and the vegetables are insulated using wheat or rice straws and stored in it for a long time. 

The comparison of crucial and general informant interviews shows an apparent decline in cited food sources and traditional cuisines. The number of citations is lower than that of the list shared by the older women. This indicates that the culture has been greatly affected due to exposure to other cultures and accessibility to the local markets. The reports of market-based food sources are prominent in general informant interviews. In contrast, home-grown and wild food resources are frequently reported by the key informants in both linguistic groups. The general informant stated that a better variety of food ingredients is now available in the markets, so plant foraging is not practiced. Most general informants preferred contemporary culinary practices due to the convenience and facilities that make cooking easy. Moreover, one of the respondents in Ragh village of Rech said introducing tools like pressure cookers has made cooking easier than in old times. Most of the general informants in Rech do not prefer traditional cuisines due to slow cooking because the time consumed in slow cooking can be utilized in other educational activities. Due to differences in lifestyle, especially educational activities, the interest in the young generation is fading. In contrast, the trend of citations relevant to animal-based food sources is much better than that of plants. More than 80% (approx.) of the general informants mentioned dairy products as a significant part of the local food system. 

The significance of women in the traditional food system emphasizes the relationship between gender, culture, and food security. Previous studies [28,41,51] have indicated that women’s participation in food systems is critical to their sustainability and resilience. Thus, valuing and promoting women and their traditional knowledge is essential to achieving long-term food security and fostering sustainable development.

The traditional knowledge possessed by Wakhi and Kho women has significantly increased diet diversity and nutritional sufficiency, which are significant pillars of food security. Women’s conventional cooking methods use locally produced foods to support sustainable agriculture practices and reduce dependency on outside food sources. These women preserve and transmit traditional food knowledge, which helps to maintain biodiversity and cultural legacy, contributing to sustainable food systems.

### 3.8. Threats to Traditional Gastronomy of Chitral and Mitigation Strategies 

The study emphasized the revitalization of the TEK of food resources in the mountain area due to the global trend toward the organic way of life.

- The study area has observed the temporary migration of youth for education and income-generation activities, which is causing rapid cultural blending and less interest in traditional ways of living. Thus, the level of rapid modernization and changing lifestyles in the young generation is causing the cultural integrity of traditional foods to fade.
- Approximately 85% of the respondents identified the younger generation’s detachment from cultural heritage and natural resources, attributing it to multiple socio-economic factors. The youth find traditional practices like foraging outdated and useless, as they can easily access the nearby markets and purchase contemporary food items even though it requires mental, physical, and financial effort.
- Most of the young Wakhi people were aware about the local name of plant species in Khowar instead of traditional Wakhi names. This can be attributed to the dominance of Kho in the region and the intermarriages between the two linguistic groups. But it is noteworthy to preserve the Wakhi language and heritage.
- The reports of market-based food sources are prominent in general informants, while home-grown and wild food resources were frequently reported by the key informants in both linguistic groups. According to the general informants, most of the food ingredients are easily available in the local markets, so the foraging of food plants is limited. Moreover, they preferred contemporary culinary practices due to the convenience and facilities that make cooking easy. For instance, one of the respondents in Ragh village of Rech valley mentioned that the use of modern facilities like pressure cookers has made cooking easier compared to old times.
- In contrast, the trend of citations relevant to animal-based food sources is in much better shape compared to food plants. More than 80% of the general informants mentioned dairy products as an important component of the local food system, specifically in Broghil. However, the increasing flux of tourists in the region rapidly damages the natural settlement. For example, the extensive use of motorbikes and other means of transport pollutes and disturbs the ecosystem and affects the natural wild animals. However, due to increasing tourism, the locals have initiated service provision in the area. However, the development of the hospitality business in the region is beneficial but not at the cost of environmental loss. The construction of buildings can be threatening to the local environment. Therefore, timely, inclusive responses to under-managed tourism and development in the study area must be tackled for a secure future.

Some suggestions based on the observation mentioned above to mitigate the threats to traditional gastronomy of Chitral are as follows.

- Community-led projects that document information sharing between younger and older generations to ensure the preservation and conservation of food traditions should be launched in the study area and its allied regions.
- Local, national, and international organizations must create entrepreneurship opportunities for youth and encourage them to create fusion by combining traditional food products with contemporary culinary art and innovative recipes from traditional cuisines.
- Promoting traditional gastronomy through food tourism and food business should elevate the socioeconomic standing of the local populations. Traditional cuisines and food culture must also be revitalized through local food festivals and events.
- Lastly, youth must be educated by integrating food culture education and its associated health benefits into the community education system and initiatives. Community organizations must engage youth in preserving food resources and bio-cultural diversity through digital campaigns employing social media platforms in the best possible ways and partnering with local stakeholders.

## 4. Conclusions

Our findings revealed that Wakhi and Kho women have deep roots in their food culture, and as the identifiers, collectors, preservers, and servers of food, they are the key contributors to household food security and sustainability of food systems in upper Chitral. Specifically, in the high alpine pastures of Broghil, diverse types of dairy products and animal-based traditional cuisines prepared by women are essential elements for the survival of Wakhi pastoralists. The degree of heterogeneity in traditional gastronomy is attributed to the ecotone difference between Wakhi and Kho communities, but intermarriages and communal interaction exhibited a slight homogeneity. Moreover, the possession of indigenous knowledge is poorly replicated among young females due to changing lifestyles, outmigration from the valleys for education, and changes in food behaviors due to the invasion of commodified food ingredients. The study findings emphasize how vital indigenous women are to preserving and sustaining local and regional food security and the important role they play in resource management, resilience building, and sustainable agriculture. Ultimately, the findings of this study can help design more inclusive and successful strategies for promoting sustainable development and increasing food security in a range of cultural contexts throughout the world. Therefore, a more comprehensive study must be conducted on the roles played by women in mountain agropastoral practices, household food security, and climate change mitigation, specifically to protect and preserve mountain food resources. In addition, a right policy action is required to revitalize the understanding of local flora, and fauna used in the food system. Most importantly, women-centered traditional food knowledge must be considered in policies framework, especially their marginal status within these communities, considering the fact that finding viable opportunities to bring the women of social inequalities related to managing food security and sustainable food systems.

## Figures and Tables

**Table 1:** Characteristics of the study area and study participants.

Sites	Veg.	LG	SA	MG	EG	Villages	GA	Elev. (m.a.s.l)	HH	NP. (KI/GI)	Age	Occupation
Rech valley	Alpine, Sub-alpine	Khowar	Farming, animal husbandry	Exogamic	Sunni & Ismaili	Nisur	Mountains and valleys	2625	192	3/5	15–80	Housewife, Teachers, Health Worker, Students
						Murech		2678	164	3/5		Housewife, Teachers
						Sorech		2770	205	3/5		Health Worker, Students
						Ragh		2712	115	3/5		Housewife, Teachers
						Phurgram		2910	125	3/5		Health Worker, Students
Broghil valley	Alpine, Sub-alpine	Wakhi	Animal husbandry, Pastoralism	Exogamic	Ismaili	Garam Chashma	Mountains, Glaciers, Pastures, wetland	3349	56	4/4		Housewife, Students
						Chikar		3629	32	3/4		Housewife, Students
						Chilmar Abad		3552	33	2/5		Housewife, Students
						Ishkarwaz		3510	27	2/3		Housewife, Students
						Lashkargaz		3708	43	3/5		Housewife, Students

Key: Veg, vegetation; LG, linguistic group; SA, subsistence activities; MG, marriage; EG, ethnic group; GA, geographical attributes; Elev, elevation; HH, household; NP, number of participants; KI, key informants; GI, general informants.

**Table 2:** Food plant diversity and utilization among Kho and Wakhi communities of upper Chitral.

S. #	Scientific Name/Family/Voucher Number	Linguistic Group		Habit	W/C	Part Use	Food Category	RFC	URs (Kho)	URs (Wakhi)	PRF
1.	*Allium barsczewskii* Lipsky Amaryllidaceae CUHA-407	Kach	Kach	H	W	Ap	Vegetable/Salad	0.853	33	31	[14,32]
2.	*Allium carolinianum* Redoutè Amaryllidaceae CUHA-115	Latruk	Lanturk	H	W	Ap	Vegetable	0.760	23	34	[28]
3.	*Allium cepa* L. Amaryllidaceae CUHA-408	Threshtu	Piyoz	H	C	Wp	Cooked/Raw	0.453	3	56	[33]
4.	*Anthemis nabataea* Eig Asteraceae CUHA-409	Shirvisht	Shirvishun	H	W	Fl	Tea/flavoring/spice	0.373	37	9	[34]
5.	*Artemisia absinthium* L. Asteraceae CUHA-410	Kharkhalich		H	W	Sd	Tea/Soup	0.775	43		[35]
6.	*Atriplex hortensis* L. Amaranthaceae CUHA-411	Dar Kunakh		H	W/C	Ap	Vegetable	0.725	23		
7.	*Benincasa fistulosa* (Stocks) H.Schaef. and S.S.Renner Cucarbitaceae CUHA-412	Tinda		Cl	C	Frt	Vegetable	0.475	19		[36]
8.	*Berberis vulgaris* L. Berberidaceae CUHA-413	Chowenj	Zolg	S	W	Ap	Snack	0.293	14	8	[28]
9.	*Beta vulgaris* L. Amaranthaceae CUHA-414	Lablabu		H	C	Rt	Salad/Snack	0.875	38		[37]
10.	*Brassica napus* L. Brassicaceae CUHA-415	Kalam		S	C	Rt	Vegetable/Snack	0.825	40		[37]
11.	*Brassica oleracea* L. Brassicaceae CUHA-416	Gobi		H	C	Lvs	Vegetable	0.075	03		[37]
12.	*Brassica rapa* L. Brassicaceae CUHA-417		Chirogh	H	W	Lvs	Vegetable	0.171		06	[14]
13.	*Buglossoides arvensis* (L.) I.M.Johnst. Boraginaceae CUHA-418	Shaghechi		H	W	Ap	Vegetable	0.075	03		[38]
14.	*Capparis spinosa* (Lam.) Capparaceae CUHA-419	Kaveer		S	W	Fl	Vegetable/Soup/Condiment	0.525	21		[28]
15.	*Carthamus tinctorius* L. Asteraceae CUHA-420	Pom		H	C	Fl	Food Color	0.300	24		
16.	*Cenchrus americanus* L. Morrone Poaceae CUHA-421	Grass		H	C	Grn	Cereal	0.275	11		[39]
17.	*Chenopodium album* L. Amaranthaceae CUHA-423	Kunakh	Shlieet	H	W/C	Ap	Vegetable	0.453		26	[14]
18.	*Chenopodium foliosum* Asch. Amaranthaceae CUHA-422		Shittet	H	W	Lvs/Frt	Vegetable/Snack	0.057		02	[28]
19.	*Coriandrum sativum* L. Apiaceae CUHA-424	Dhanu	Dhanu	H	C	Ap	Condiment	0.520	43	8	[35]
20.	*Cucurbita maxima* Duchesne CucurbitaceaeCUHA-425	Alok		Cl	C	Frt, Sd	Vegetable	0.200	10		[37]
21.	*Cuminum cyminum* L. Apiaceae CUHA-426	Zeera	Zeera	H	W	Sd	Tea/Condiment	0.653	57	16	[37]
22.	*Daucus carota* L. Apiaceae CUHA-427	Kheshgoom	Zarduk	H	C	Rt	Vegetable/Salad	0.560	61	8	[34,35]
23.	*Eremurus stenophyllus* Baker Asphodelaceae CUHA-428	Laqanch	Laq	H	W	Ap	Vegetable	0.880	33	37	[14]
24.	*Ferula narthex* L. Apiaceae CUHA-429	Rauw		H	W	Sht	Snack	0.375	15		[2,34]
25.	*Hippophae rhamnoides* L. Elaeagnaceae CUHA-430	Khilghitu	Zakh Khusgik	S	W	Frt	Snack	0.066	2	3	[14]
26.	*Hordeum vulgare* L. Poaceae CUHA-431	Siri	Yirk	H	C	Grn	Cereal	0.680	21	30	[37]
27.	*Hylotelephium* Spp. Crassulaceae CUHA-432	Asqarbash		H	W	Ap	Vegetable	0.375	15		[40]
28.	*Juglans regia* L. Juglandaceae CUHA-45	Birmough		T	C	Nt	Snack/oil/Flavoring	0.700	65		[28,34]
29.	*Lactuca sativa* L. Asteraceae CUHA-433	Kileem	Kileem	H	C	Ap	Salad	0.226	16	1	[37]
30.	*Lathyrus oleraceus* Lam. Fabaceae CUHA-434	Mater		H	C	Sd	Vegetable	0.425	17		[36]
31.	*Lens culinaris* MediK Fabaceae CUHA-435	Sirju		H	C	Sd	Vegetable/Flour	0.500	39		[37]
32.	*Lepidium ruderale* Hk. & Anders Brassicaceae CUHA-436	Palak khardachi		H	C	Ap	Salad	0.100	4		[37]
33.	*Lepyrodiclis holosteoides* (C.A.Mey.) Caryophyllaceae CUHA-437	Birghal	Yorkwush	H	W	Ap	Vegetable	0.747	29	27	[14]
34.	*Malus domestica* (Suckow) Borkh. Rosaceae CUHA-438	Palough		T	C	Frt	Fruit	0.425	29		[28]
35.	*Malva neglecta* Wallr. Malvaceae CUHA-286	Suachal	Suachal	H	W	Ap	Vegetable	0.067	2	3	[28]
36.	*Medicago sativa* subsp. *Sativa* (L.) Fabaceae CUHA-440	Lalmi		H	W/C	Ap	Vegetable	0.100	4		[28]
37.	*Mentha longifolia* (L.) Huds. Lamiaceae CUHA-48	Bain		H	W	Lvs	Tea/Salad	0.225	14		[35]
38.	*Mentha suaveolens* Ehrh. Lamiaceae CUHA-441	Pudina		H	W/C	Ap	Tea/Condiment	0.075	6		[37]
39.	*Nasturtium officinale* R.Br. Brassicaceae CUHA-50	Troqkhard achi		H	W	Ap	Salad	0.075	3		[31]
40.	*Panicum miliaceum* L. Poaceae CUHA-55	Olien		H	C	Grn	Cereal	0.375	15		[37]
41.	*Papaver involucratum* Popov. Papaveraceae CUHA-442		Gulmorwoi	H	W	Lvs	Tea	0.343		12	[14]
42.	*Pisum sativum* L. Fabaceae CUHA-443	Kuchoon	Sakh	Cl	C	Sd	Lentil/Snack/Flour	0.587	9	11	[37]
43.	*Prunus amygdalus* Batsc. Rosaceae CUHA-444	Badam		Tree	C	Nt	Fruit	0.125	5		[35]
44.	*Prunus armeniaca* L. Rosaceae CUHA-73	Zhuli		T	C	Frt, Nt	Fruit	0.425	34		[35]
45.	*Prunus avium* L Rosaceae CUHA-445	Cherry		T	C	Frt	Fruit	0.050	2		[41]
46.	*Pyrus bourgaeana* Decne. Rosaceae CUHA-445	Tong		T	C	Frt	Fruit	0.275	15		[41]
47.	*Raphanus raphanistrum* (L.) Brassicaceae CUHA-447	Shalmo	Sholum	H	C	Wp	Vegetable/Salad	0.680	53	23	[37]
48.	*Rheum ribes* L. Polygonaceae CUHA-448	Ishpar	Ishpat	H	W	Sht	Snack	0.173	11	2	[28]
49.	*Rumex dentatus* Rech.f. Polygonaceae CUHA-91	Chirkonjur	Shilkha	H	W	Ap	Vegetable	0.587	18	26	[28]
50.	*Salvia rhytidea* Benth Lamiaceae CUHA-449	Korotch		H	W	Ap	Salad/Condiment	0.150	9		[42]
51.	*Saxifraga sibirica* L. Saxifragaceae CUHA-450	Dromosuru		H	W	Sht	Condiment	0.350	14		
52.	*Silene conoidea* L. Caryophyllaceae CUHA-324	Apupar		H	W	Ap	Vegetable	0.625	25		[28]
53.	*Solanum lycopersicum* L. Solanaceae CUHA-451	Patingal		H	C	Frt	Salad/condiment	0.300	17		[37]
54.	*Solanum melongena* L. Solanaceae CUHA-452	Patigan		H	C	Frt	Vegetable	0.025	1		[37]
55.	*Solanum tuberosum* L. Solanaceae CUHA-453	Alu	Olue	H	C	Tbr	Vegetable/Snack	0.613	44	17	[37]
56.	*Taraxacum campylodes* G.E.Haglund Asteraceae CUHA-336		Pops	H	W	Lvs	Tea	0.143		5	[14]
57.	*Thymus serpyllum* L. Lamiaceae CUHA-454		Jimbilak	H	W	Lvs	Tea	0.600		21	[14]
58.	*Trigonella gladiata* Steven ex M.Bieb. Fabaceae CUHA-455	Sugon		H	W	Ap	Condiment	0.125	6		[35,42]
59.	*Triticum aestivum* subsp. *spelta* (L.) Thell. Poaceae CUHA-456	Gom	Ghidim	H	C	Grn	Cereal	0.680	35	16	[37]
60.	*Vicia faba* L. Fabaceae CUHA-457	Andalo		H	C	Sd	Snack	0.100	6		[37]
61.	*Vigna unguiculata* (L.) Walp. Fabaceae CUHA-458	Lobia		Cl	C	Sd	Lentil	0.200	8		[37]
62.	*Zataria multiflora* Boiss. Lamiaceae CUHA-459	Troshnagholi		H	W/C	Ap	Condiment	0.050	4		[42]
63.	*Zea mays* subsp. *mays* L. Poaceae CUHA-460	Juari		H	C	Sd	Cereal	0.850	44		[35]
64.	*Ziziphora clinopodioides* (Rech.f.) Lamiaceae CUHA-461	Xhughur		H	W	Ap	Condiment	0.025	1		[14]
65.	*Zygophyllum obliquum* Popov Zygophyllaceae CUHA-462		Yumwush	H	W	Lvs	Vegetable	0.400		14	[14]

H, Herb; S, Shrub; T, Tree; Cl, Cimber; Ap, Aerial part; Sd, Seed; Lvs, Leaves; Grn, Grains; Fl, Flower; Rt, Root; Nt, Nut; Wp, Whole plant; Sht, Shoot; Frt, Fruit; RFC, Relative frequency of citation; URs, Use reports; PRF: Previously reported as food; W/C, Wild/cultivated.

**Table 3:** Animal-based food products used by Kho and Wakhi communities.

S. #	Scientific Name/Family	Habit	Local Name		Part(s) Used	Products	RFC
			Khowar	Wakhi			
1.	*Anas crecca* L. Anatidae	Wild	Alhi	Yoch	Meat	Pushur ^K^, Ghusht ^W^	0.280
2.	*Bos grunniens* L. Bovidae	Domesticated	Zogh	Zugh	Milk	Mrik ^W^	0.747
						Xarj ^W^	0.653
						Rugon ^W^	0.587
						Digh ^W^	0.533
						Phai ^W^	0.480
						Khomrugon ^W^	0.387
					Meat	Pushur ^K^, Ghusht ^W^	0.280
					Fat	Zagh ^K^, Zigh ^W^	0.173
3.	*Bos taurus* L. Bovidae	Domesticated	Leshu	Ghev	Milk	Phenak ^K^	0.750
						Trin ^K^	0.750
						Khombokh ^K^, Mrik ^W^	0.747
						Maska ^K^, Khomrugon ^W^	0.387
						Qurut ^W^	0.657
						Cheer ^K^, Xarj ^W^	0.653
						Don ^K^, Rugon ^W^	0.587
						Shetu ^K^, Digh ^W^	0.533
						Macheer ^K^, Phai ^W^	0.480
						Shiphinak ^K^	0.450
						Phaneer ^W^	0.400
						Shut ^K^	0.375
						Chaka ^K^	0.025
					Meat	Pushur ^K^, Ghusht ^W^	0.280
					Fat	Zagh ^K^, Zigh ^W^	0.173
4.	*Capra hircus* L. Bovidae	Domesticated	Pai	Toogh	Milk	Phenak ^K^	0.750
						Trin ^K^	0.750
						Khombokh ^K^, Mrik ^W^	0.747
						Maska ^K^, Khomrugon ^W^	0.387
						Qurut ^W^	0.657
						Cheer ^K^, Xarj ^W^	0.653
						Don ^K^, Rugon ^W^	0.587
						Shetu ^K^, Digh ^W^	0.533
						Macheer ^K^, Phai ^W^	0.480
						Shiphinak ^K^	0.450
						Phaneer ^W^	0.400
						Shut ^K^	0.375
						Chaka ^K^	0.025
					Meat	Pushur ^K^, Ghusht ^W^	0.280
					Fat	Zagh ^K^, Zigh ^W^	0.173
5.	*Capra sibirica* Pallas Bovidae	Wild	Tonishu	Jondor	Meat	Pushur ^K^, Ghusht ^W^	0.280
					Fat	Zagh ^K^, Zigh ^W^	0.173
6.	*Columba leuconota* Vig. Columbidae	Wild		Kebet	Meat	Ghusht ^W^	0.280
7.	*Columba livia* Gmelin Columbidae	Wild	Aghagh		Meat	Pushur ^K^	0.280
8.	*Gallus domesticus* L. Phasianidae	Domesticated	Kahak	Karkh	Eggs	Ayukun ^K^	0.307
					Meat	Pushur ^K^	0.280
9.	*Ovis arie* L. Bovidae	Domesticated	Keli	Maye	Meat	Pushur ^K^, Ghusht ^W^	0.280
					Fat	Zagh ^K^, Zigh ^W^	0.173
10.	*Streptopelia decaocto* Friv. Columbidae	Wild	Kalkor	Felanch	Meat	Pushur ^K^, Ghusht ^W^	0.280

Key: K, Khowar; W, Wakhi; RFC, relative frequency of citation.

**Table 4:** Traditional cuisines prepared by Kho and Wakhi women using local plants and animal-based ingredients.

S. #	Khowar	Wakhi	Major Ingredients	Recipes	Source	RFC
1.	Andalai		Kuchoon (Peas) + Barley (Siri)	Pea and barley flour is mixed at an equal ratio, and dough is prepared with salt to taste. A flatbread is then baked.	Plant	0.125
2.	Atala		Wormwood + Cumin seeds + Butter + Salt	The seeds are fried in butter/ghee, and flour is added along with water and simmered until done. Salt is added as per the requirement	Plant	0.225
3.	Brat	Qomuchduni	Flour + Butter/Ghee + Salt	The dough is kneaded, puffed, and then flattened to make flat bread. Oil/ghee is used to polish either side of the bread, and it is baked in the traditional way using coal and ashes.	Plant	0.627
4.	Chambor Tiki		Dried apricot + Apricot kernels	Dried apricots are ground and shaped with apricot kernels. Eaten as a snack.	Plant	0.025
5.	Cheer Grinj	Sheerbronj	Milk + Rice + Salt	Rice is boiled in water with salt to taste. When it is tender enough, milk is added. The dish is simmered until it has a uniform texture and consistency.	Plant + Animal	0.747
6.	Cheera Kali		Milk + Salt + Flour	Milk is boiled with salt to taste. Flatbread is rolled out and cut into noodles or fine pieces. The pieces are added to the milk and boiled until done.	Plant + Animal	0.550
7.	Cheera Leganu	Xarlmoch	Wheat flour + Milk +Salt	The wheat flour is turned into small dough balls using the traditional method, then boiled in milk with salt until soft.	Plant + Animal	0.280
8.	Cheera Mul	Xarjmul	Wheat flour + Milk +Salt	Wheat flour is added to boiling water with salt to taste. The dish is gradually heated and boiled until done. In another pot, milk/cream is boiled and added to the dish, which is then served.	Plant + Animal	0.373
9.	Cheera Shapik	Xarjkhech	Milk + Wheat flour + Salt + Butter/Ghee	Milk is heated in a pot, seasoned with salt according to taste, and brought to a boil. A small amount of flour is added to thicken the milk. After getting to a certain texture, the paste is cooled. On the other side, flatbread, locally called Phulka, is prepared. The paste is sandwiched between flat bread or tortillas. The dish is topped either with the same paste or butter.	Plant + Animal	0.653
10.	Chuchu Shoshp		Shoshp Peshiru (Sweetened wheat flour) + Milk + Desi ghee	Water is boiled, added to sweetened wheat flour, and simmered until done. It is served with milk, desi ghee, or walnut oil.	Plant + Animal	0.375
11.	Ghalmandi		Cottage cheese + Coriander +Salt + Butter +Tortilla	Cottage cheese, coriander, and salt are added, and a paste is prepared. On the other side, flatbread, locally called Phulka, is prepared. The paste is sandwiched between flat bread or tortillas. The dish is topped with sizzling butter.	Plant + Animal	0.875
12.	Ghara		Millet + Salt + Milk	The millet is finely ground and boiled in water with salt to taste until it softens. Then, milk is added and boiled until it has the desired consistency and flavor.	Plant	0.075
13.	Kali	Osh	Onions + Green Spices +Cumin + Caper + Meat + Flatbread	Sliced onions are fried in oil and added to some flavoring and spice. The most used flavoring agents are cumin, caper, and meat. After frying the spices, the water is heated until it boils. Thin strips of dough are sliced and added to the oiling stew. The stew then simmered until done.	Plant	0.413
14.	Kaveerogh		Caper +Meat	Caper flowers are boiled with the meat stew until develop flavor.	Plant	0.425
15.	Khaigina		Eggs + Milk +Salt	Milk is boiled and added to whisked eggs and salt; later, hard-boiled eggs are also added.	Plant + Animal	0.075
16.	Khesta Shapik	Khestakhech	Wheat flour + Yeast	A liquid dough is prepared and kept until it poofs; then, flatbread is prepared and toasted.	Plant + Animal	0.787
17.	Khisht		Wheat flour + Desi ghee + Salt	Desi ghee/butter is melted and added to flour and salt and thoroughly mixed. The mixture is fried and then shaped into bread by pressing.	Plant + Animal	0.200
18.	Kotbat		Milk + Rice +Desi ghee + Flour	This is a combination of two traditional dishes namely Sanabachi and Cheergrinj. The Cheergrinj is topped with Sanabachi.	Plant + Animal	0.600
19.	Kuchunai	Sakhech	Dried peas +Salt	The local variety of peas is dried and ground. The flour is then kneaded, and thick flatbread is toasted/griddled.	Plant	0.093
20.	Laxek		Wheat grain + Salt + Meat	Soaked wheat grains are ground well. Meat is boiled until tender; salt is added for taste, and later, the ground wheat grains are added to the meat stew.	Plant + Animal	0.625
21.	Leganu	Moch	Lentil flour + Tomato + Onion, Green seasoning + Salt	Lentil flour is turned into small dough balls using the traditional method and then boiled in water with salt it until softens. In a frying pan, onion, tomatoes, and green spices and seasoning are fried and seasoned on top of it and left to rest for a while.	Plant + Animal	0.775
22.	Leganu Muxhi		Leganu (Cuisine 21) + Flour + Salt	This is a combination of two traditional dishes; Leganu is sandwiched between two Khesta Shapik (fermented flat roti) and toasted.	Plant	0.025
23.	Mul	Mul	Wheat flour + Salt + Butter	Wheat flour is added to boiling water with salt to taste. The dish is gradually heated and boiled until done. Sizzling butter/desi ghee is seasoned on top of the dish and served.	Plant	0.560
24.	Mulida	Molda	Milk, Salt, Desi Ghee, Flour	Milk is boiled with salt to taste, and boiled milk is added to already toasted tortilla pieces. Later it is seasoned with butter/desi ghee	Plant + Animal	0.280
25.	Pakhti	Bronj	Rice + Salt + Ghee/Oil	Water with salt to taste is boiled. Already soaked rice is added to boiling water and cooked until the water evaporates. A deep-heated oil/ghee is seasoned on top of the rice.	Plant	0.453
26.	Phenak Paratha		Cottage cheese + Flour + Salt	After the flour is kneaded, it is flattened in a round shape. A paste is prepared with cottage cheese, salt, and green seasonings. The flattened bread is filled with the paste and then griddled/toasted or fried.	Plant and Animal	0.125
27.	Qalaibat		Animal fat +Flour+ Salt,	After heating, fat is mixed with equal amounts of water and salt. Later, wheat flour is added, and the dish is stirred and simmered until done.	Plant + Animal	0.325
28.	Rishok		Flour +Eggs + Salt	Flour is added to salt, egg, and water. A liquid paste is prepared. The pasted is flattened on a heated pan and toasted with oil.	Plant + Animal	0.525
29.	Sanabachi	Bat	Butter + Desi ghee + Flour + Salt + Coloring agent.	Butter/desi ghee is heated in a pot, and then flour is fried. When a light brown color appears, a coloring agent (safflower) is mixed to enhance color. Then, water is added and cooked until it attains the required consistency.	Animal	0.920
30.	Sanabachi Tiki		Sanabachi (Cuisine 29) + Flour + Salt + Oil	The sanabachi and cottage cheese are filled in bread, and the bread is baked traditionally.	Plant + Animal	0.375
31.	Shakarpostek		Dried mulberries +Walnuts	Dried mulberries are ground traditionally and added to walnuts. The mix is further pounded until a desired texture and used as a dessert.	Plant	0.075
32.	Shakh		Green vegetable + Salt + Tomatoes + Onion + Spices + Oil	The vegetable is boiled and mashed into a paste. In another pot, gravy is prepared with onions, tomatoes, salt, and other spices. The vegetable paste is added to the gravy and fried. A certain amount of water is also added. When it boils a flour paste is added. Then the dish is simmered until done.	Plant	0.800
33.	Shakh Muxhi		Cooked vegetable (Sakh) + Khesta Shapik (Cuisine # 16)	The cooked vegetable (Sakh) is sandwiched between two pieces of Khesta shapik.	Plant	0.300
34.	Shapiko Sora Don	Rugonkhech	Flat bread + Desi ghee	A flatbread is seasoned with hot sizzling desi ghee and served.	Plant + Animal	0.253
35.	Shorab Chai		Tea + Salt + Walnuts + Milk	Water is boiled with tea bags, salt, and crushed walnuts. After a good boil, milk is added and simmered until it develops a taste.	Plant + Animal	0.075
36.	Shoshp	Shushp	Shoshp Peshiru (Sweetened wheat flour) + Walnut	Walnuts are heated until produce oil; the walnut oil is added to water. When the water boils, the sweetened wheat flour is added and slow cooked for almost 12 h.	Plant	0.480
37.	Shoshp Kali		Shoshp Peshiru (Sweetened wheat flour) + Water	The sweetened wheat flour is added to boiling water and simmered until it attains the required consistency.	Plant	0.350
38.	Shoshp Muxhi		Shoshp + Khesta shapik	This is a combination of two traditional dishes; Shoshp is sandwiched between two Khesta shapik (fermented flat roti) and toasted.	Plant	0.050
39.	Shoshp Shapik		Flour (Sweetened) + Wheat flour	Sweetened flour is added to normal wheat flour and kneaded. Then, flatbread is prepared and toasted.	Plant	0.100
40.	Shula		Meat + Rice + Salt	The meat is boiled until it is tender, and salt is added for taste. The meat stew is added to a local variety of rice. The whole porridge is cooked until it attains required consistency.	Plant + Animal	0.240
41.	Siriae	Yirkkhech	Barley flour + Wheat grains	The flour (barley) is kneaded with a small amount of wheat. Then, flatbread is prepared and toasted.	Plant	0.240
42.	Sormuliogh		Fresh wheat aerial parts + Onion + Spices + Flavoring agent	Green wheat grass is collected from the field, and the kernels are separated using the traditional method. The grains are fried and then boiled in water until softened. In a pan, onion, green spices, and flavoring agents are fried. The fried mixture is seasoned on the top of the dish.	Plant	0.075
43.	Tarbat		Animal fat +Sweetened flour,	Animal fats are melted, and water is added to it. Later, sweetened wheat flour is added and slowly cooked until done.	Plant + Animal	0.325
44.	Tawa ShoshpShalan		Shoshp Peshiru (Sweetened Wheat Flour) + Water	The sweetened wheat flour is turned into a liquid paste and is toasted and cooked on Tawa.	Plant	0.025
45.	Thathori		Flour (Maize, Barley, Wheat) + Salt	Flour (maiz, barley) is kneaded with a small amount of wheat flour. The puffed dough is rolled out into small-size breads and griddled/toasted.	Plant	0.125
46.	Troq Muxhi		Flour +Apricot nuts + Cottage cheese	After the flour is kneaded, it is flattened into a round shape. A paste is prepared with crushed apricot nuts and cottage cheese with salt and other green seasonings in it. The flattened bread is filled with the paste and then griddled/toasted.	Plant	0.025
47.	Xholai Brat		Flour + Walnuts + Potatoes + Onion + Salt + Other Seasonings + Desi ghee/Oil	The dough is kneaded when it is puffed then flattened to make flat bread. A paste is prepared with crushed walnuts, boiled potatoes, sliced onions, and salt. The paste is used to fill in the bread, oil/ghee is used to polish either side of the bread, and the bread is baked in the traditional way using coal and ashes.	Plant	0.525
48.	Xholai Shapik		Flour + Walnuts + Potatoes, Onion + Salt +Other Seasonings	A dough is kneaded and flattened to make flatbread. A paste is prepared with crushed walnuts, boiled potatoes, sliced onions, and salt. The paste is used to fill in the bread, and it is toasted/griddled.	Plant	0.050
49.		Qurutaab	Qurut (Cheese) + Bread + Desi ghee	Qurut is soaked in water until it softens, and pieces of flatbread are added to this liquid/juice and seasoned with desi ghee.	Animal	0.743
50.		Thonikcha	Desi ghee + Flour + Salt + Milk + Qurutogh (Qurut water)	Flour with desi ghee, salt, and milk is kneaded, and the dough is prepared. The flattened bread is pasted in a clay oven, and milk or Qurutogh is splashed on it and toasted.	Plant + Animal	0.657
51.		Shindet	Flatbread + Desi ghee + Milk	A pre-toasted bread is soaked in milk and seasoned with sizzling desi ghee.	Plant + Animal	0.371
52.		Khishkphai	Oil +Flour + Salt	1: After heating some oil, flour is added and cooked until it turns brown. After that, water is added and cooked until finished, and salt is added for taste. 2: milk is boiled with salt in it. Flour is added and simmered until done.	Animal	0.057
53.		Ptoq	Flour +Salt + Yeast + Qurutogh/Milk	Flour is kneaded with salt, and when it is puffed, bread is rolled out. The rolled-out small breads are pasted in the clay oven with a splash of Qurutogh and milk and griddled/toasted.	Plant + Animal	0.600
54.		Objush	Milk + Salt + Water	Water is boiled, and milk and a pinch of salt are added to taste. Then, the solution is boiled for some time and drank as tea.	Animal	0.086
55.		Dildongi	Flour + Salt + Yeast	Flour is kneaded with salt, and when it is puffed, bread is rolled out. The rolled-out bread is pasted in the clay oven and griddled/toasted.	Plant + Animal	0.686
56.		Jigoe	Milk + Phai (Curd)	Milk is boiled, and Phai is added and boiled until the required consistency is attained.	Animal	0.057

## Data Availability

All data are included in the manuscript; however, for additional information, the corresponding author may be consulted.
